# Subnormal Peripheral Blood Leukocyte Counts Are Related to the Lowest Prevalence and Incidence of Metabolic Syndrome: Tianjin Chronic Low-Grade Systemic Inflammation and Health Cohort Study

**DOI:** 10.1155/2014/412386

**Published:** 2014-04-29

**Authors:** Shaomei Sun, Hongmei Wu, Qing Zhang, Chongjin Wang, Yinting Guo, Huanmin Du, Li Liu, Qiyu Jia, Xing Wang, Kun Song, Kaijun Niu

**Affiliations:** ^1^Health Management Centre, Tianjin Medical University General Hospital, Tianjin, China; ^2^Nutritional Epidemiology Institute and School of Public Health, Tianjin Medical University, 22 Qixiangtai Road, Heping District, Tianjin 300070, China

## Abstract

Few studies have assessed the relationship between a subnormal inflammatory status and metabolic syndrome (MS). We therefore designed a cross-sectional and 5-year cohort study to evaluate how a subnormal peripheral blood leukocyte count is related to MS. Participants were recruited from Tianjin Medical University General Hospital-Health Management Centre. Both a baseline cross-sectional (*n* = 46,179) and a prospective assessment (*n* = 13,061) were performed. Participants without a history of MS were followed up for 5 years. Leukocyte counts and MS components were assessed at baseline and yearly during the follow-up. Adjusted logistic and Cox proportional hazards regression models were used to assess relationships between the categories of leukocyte counts and MS. The subnormal leukocyte counts group (1,100–3,900 cells/mm^3^) had the lowest prevalence and incidence of MS. The odds ratio and hazard ratio (95% confidence interval) of the highest leukocyte counts were 1.98 (1.57–2.49) and 1.50 (1.22–1.84) (both *P* for trend <0.0001), respectively, when compared to the subnormal leukocyte counts group after adjusting for potential confounders. This study has shown that subnormal leukocyte counts are independently related to the lowest prevalence and incidence of MS. The findings suggest that it is necessary to restudy and discuss the clinical or preventive value of subnormal leukocyte counts.

## 1. Introduction

Chronic diseases, such as cardiovascular diseases (CVD), cancer, and age-related diseases, have long been considered among the most important global public health issues [1]. Clarifying the common pathological process of these diseases or statuses is a crucial step toward providing early prevention and treatment. A growing body of evidence indicates that chronic low-grade inflammation is a common pathological process and an important contributing factor to these diseases or statuses [2–4].

Various inflammatory markers, including soluble adhesion molecules (e.g., E-selectin, P-selectin, intracellular adhesion molecule-1, and vascular cell adhesion molecule-1), cytokines (e.g., interleukin-1*β*, -6, -8, and -10 and tumor necrosis factor-*α*), acute phase reactants (e.g., fibrinogen, serum amyloid A protein, and high sensitive C-reactive protein [hsCRP]), and leukocyte counts, are frequently used to assess systemic inflammatory statuses [2]. Among these inflammatory markers, peripheral blood leukocyte counts are routinely measured in clinical practice and are the only cellular marker of systemic inflammation. Furthermore, compared to other inflammatory markers, only leukocyte counts were given a lower limiting value: <4,000 cells/mm^3^. However, it is noteworthy that this reference value is based only on the 95% lower limit in the general population [5].

To date, several cross-sectional [6–8] and cohort [9–11] studies have found that leukocyte counts are positively related to the prevalence and incidence of metabolic syndrome (MS), which is a well-recognized risk factor for CVD. However, most of these studies have used quartile or quintile categories of leukocyte counts to assess the relationship. Because the percentage of participants who have subnormal leukocyte counts is generally less than 8% among the general population, these studies cannot accurately evaluate the relationship between subnormal leukocyte counts and MS among an apparently healthy population. Further, one cohort study limited leukocyte counts from 4,000 to 10,000 cells/mm^3^ during assessment [12]. It is, therefore, still unclear how subnormal leukocyte counts are related to MS.

Considering the above factors, we designed a cross-sectional and 5-year follow-up study to investigate how a subnormal leukocyte count is related to the prevalence and incidence of MS in apparently healthy adults.

## 2. Methods

### 2.1. Participants

Tianjin chronic low-grade systemic inflammation and health (TCLSIH) cohort study is a large prospective dynamic cohort study focusing on the relationships between chronic low-grade systemic inflammation and the health status of a population living in Tianjin, China [13]. Tianjin is a city of approximately 10.43 million inhabitants, located in the northeast of the North China Plain, facing the Bohai Sea [14]. Participants were recruited, while having a routine annual physical examination in 2007 at Tianjin Medical University General Hospital-Health Management Centre, the largest and most comprehensive physical examination center in Tianjin.

**Figure 1:**
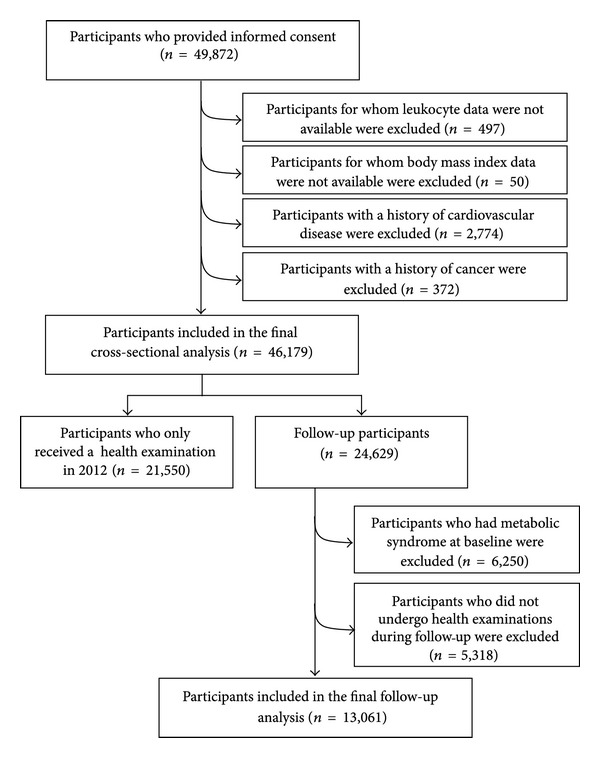
Selection of the study population, Tianjin chronic low-grade inflammation and health (TCLIH) cohort study, 2007 to 2012.

The TCLIH data from 2007 to 2012 was used in this study. The participant selection process is described in Figure 1. During the research period, there were 49,872 participants who had received at least one health examination, agreed to participate, and provided informed consent for their data to be analyzed. We excluded participants who did not have leukocyte counts (*n* = 497) or body height and/or body weight measurements (*n* = 50) or those with a history of CVD (*n* = 2,774) or cancer (*n* = 372). Owing to these exclusions, the final cross-sectional study population comprised 46,179 participants (mean standard deviation age: 45.1 12.5 years; male: 59.6%).

For follow-up analysis, participants were excluded at baseline if they had received a health examination only in 2012 (*n* = 21,550) or had MS (*n* = 6,250). 5,318 participants who did not undergo health examinations during follow-up were also excluded. Following these exclusions, the final cohort study population comprised 13,061 participants (follow-up rate: 71.1%; mean standard deviation age: 43.5 12.2 years; male: 57.2%). The protocol of our study was approved by the Institutional Review Board of the Tianjin Medical University. This study conforms to STROBE guidelines for cross-sectional and cohort studies.

### 2.2. Assessment of Leukocyte Counts

Fasting blood samples were taken by venipuncture of the cubital vein and immediately mixed with EDTA. Leukocyte and its differential counts were carried out using the automated hematology analyzer XE-2100 (Sysmex, Kobe, Japan) and expressed as ×1,000 cells/mm^3^. The test for blanks was ≤0.2 × 10^9^ cells/L; the intra- and interassay coefficients of variation (CV) were ≤2.0%; and the cross-contamination rate was ≤0.5%. In order to investigate how a subnormal leukocyte count is related to prevalence and incidence of MS, we divided participants into 8 categories according to leukocyte counts as follows: 1.1–3.9 (subnormal group), 4.0–4.9, 5.0–5.9, 6.0–6.9, 7.0–7.9, 8.0–8.9, 9.0–9.9, and ≥10.0 (high-normal group) (cells/mm^3^  ×  1,000). Furthermore, because neutrophils (50–60%) and lymphocytes (20–40%) constituted the predominant proportion of total circulating leukocytes, we also examine the relationships between quintiles of neutrophil and lymphocyte counts and MS.

### 2.3. Assessment of MS

Waist circumference was measured at the umbilical level with participants standing and breathing normally. Blood pressure (BP) was measured twice from the upper left arm using a TM-2655P automatic device (A&D Co., Tokyo, Japan) after 5 minutes of rest in a seated position. The mean of these 2 measurements was taken as the BP value. Blood samples for the analysis of fasting blood sugar (FBS) and lipids were collected in siliconized vacuum plastic tubes. FBS was measured by the glucose oxidase method, triglycerides (TG) were measured by enzymatic methods, low-density lipoprotein cholesterol (LDL) was measured by the polyvinyl sulfuric acid precipitation method, and high-density lipoprotein cholesterol (HDL) was measured by the chemical precipitation method using appropriate kits on a Cobas 8000 analyzer (Roche, Mannheim, Germany). Serum uric acid levels were determined according to a phosphotungstic acid reduction method with the Cobas 8000. Albumin in serum was measured by the bromocresol green method with the Cobas 8000. Plasma fibrinogen, which is a predictor of stroke and myocardial infarction [15], was determined by the freezing method with the autoanalyzer CA-1500 (Sysmex).

MS was defined in accordance with the criteria of the American Heart Association scientific statements of 2009 [16]. Participants were considered to have MS when they presented three or more of the following components.

- Elevated waist circumference for Chinese individuals (≥85 cm and ≥80 cm in women and men, resp.).
- Elevated TG (≥1.7 mmol/L) or drug treatment for elevated TG.
- Reduced HDL (<1.0 mmol/L in men; < 1.3 mmol/L in women) or drug treatment for reduced HDL.
- Elevated blood pressure (SBP ≥ 130 mm Hg and/or DBP ≥ 85 mm Hg) or antihypertensive drug treatment.
- Elevated fasting glucose (≥5.56 mmol/L) or drug treatment of elevated glucose.

### 2.4. Assessment of Other Variables

Anthropometric parameters (height and body weight) were recorded using a standard protocol. Body mass index (BMI) was calculated as weight/height^2^ (kg/m^2^). Sociodemographic variables, including gender, and age were also assessed. A detailed personal and family history of physical illness and current medications was noted from “yes” or “no” responses to relevant questions. Information on smoking and drinking statuses was obtained from a questionnaire survey.

### 2.5. Statistical Analysis

All statistical analyses were performed using the Statistical Analysis System version 9.3 for Windows (SAS Institute Inc., Cary, NC, USA). Descriptive data is presented as the mean (95% confidence interval, CI) for adjusted continuous variables and as percentages for categorical variables.

For analysis, the prevalence and incidence of MS were used as dependent variables, and categories of leukocyte counts and quintiles of neutrophil and lymphocyte counts were used as independent variables. For baseline characteristics analysis, the differences among leukocyte count categories were examined using analysis of covariance (ANCOVA) for continuous variables and multiple logistic regression analysis for proportional variables after adjustment for age and sex. Multiple logistic regression analysis was used to examine relationships between leukocyte count categories, quintiles of neutrophil and lymphocyte counts, and the prevalence of MS after adjustment for covariates: age, sex, baseline BMI, smoking status, drinking status, and family history of CVD, hypertension, hyperlipidemia, and diabetes. The Cox proportional hazards regression model was used to examine the relationships between leukocyte count categories, quintiles of neutrophil and lymphocyte counts, and the incidence of MS with adjustment for the covariates mentioned above.

An odds ratio (OR), hazard ratio (HR), and 95% CI were calculated. All *P* values for linear trends were calculated using the median value of leukocyte count categories or quintiles of neutrophil and lymphocyte counts. All tests were two-tailed and *P* < 0.05 was defined as statistically significant.

## 3. Results

From 2007 to 2012, prevalence of MS was 19.8, 24.7, 28.8, 29.9, 33.4, and 34.4, respectively. The percentages of participants with a subnormal leukocyte count were 7.7% and 8.6% for cross-sectional and follow-up analysis, respectively.

Age- and sex-adjusted participant characteristics in relation to leukocyte count categories for cross-sectional analysis are presented in Table 1. Compared to participants in the subnormal leukocyte counts group, those in the highest category tended to be younger and to have higher BMI, waist circumference, TC, TG, LDL, SBP, DBP, FBS, serum UA, and fibrinogen and lower HDL. A higher proportion of these participants were male, with a higher proportion of current smokers and alcohol consumers and a higher proportion of family history of hypertension and diabetes (*P* for all trends ≤0.02). Other than these results, no significant differences were observed between participants in the leukocyte count categories.

**Figure 2:**
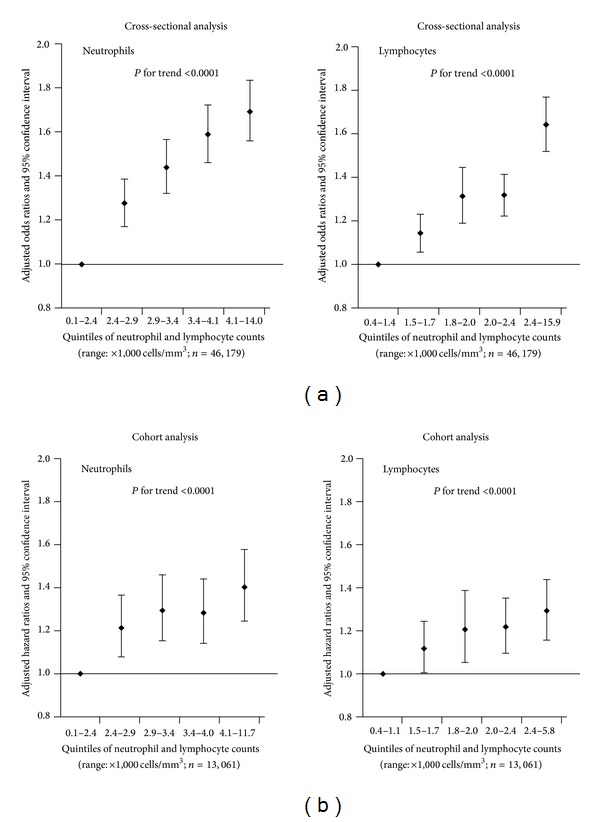
Adjusted odds ratios (a) and hazard ratios (b) (95% confidence interval) of the relationship between the quintiles of neutrophil and lymphocyte counts and metabolic syndrome. Adjusted for age, sex, BMI, smoking status, drinking status, and family history of cardiovascular disease, hypertension, hyperlipidemia, and diabetes.

The crude and adjusted relationships between leukocyte count categories and MS and its components are indicated in Table 2. In the final multivariate models, the adjusted ORs (95% CI) of MS were related to the gradual increase of the categories of leukocyte counts as compared with participants who had subnormal leukocyte counts and were as follows: 1.19 (1.06, 1.34), 1.56 (1.39, 1.74), 1.74 (1.55, 1.96), 2.02 (1.78, 2.29), 2.20 (1.90, 2.56), 2.28 (1.87, 2.77), and 1.98 (1.57, 2.49), respectively (*P* for trend <0.0001). Similar relationships were also observed between the categories of leukocyte counts and MS components. Moreover, after adjustment for potential confounders, the ORs (95% CI) of MS for increasing quintiles of neutrophil and lymphocyte counts were 1.00, 1.28 (1.18, 1.38), 1.44 (1.33, 1.56), 1.59 (1.47, 1.72), and 1.69 (1.56, 1.83) (*P* for trend <0.0001) and 1.00, 1.15 (1.06, 1.23), 1.32 (1.20, 1.45), 1.32 (1.23, 1.42), and 1.64 (1.53, 1.77) (*P* for trend <0.0001), respectively (Figure 2(a)).

Age- and sex-adjusted baseline characteristics for follow-up analysis are shown in Table 3. Because the number of participants in groups 6–8 of leukocyte counts was smaller and had essentially similar results, we combined these groups during subsequent statistical analysis. The baseline results were similar to the participant characteristics in cross-sectional analysis, with the exception of FBS and the proportion of those with a family history of hypertension. No significant differences were observed between the categories of leukocyte counts, FBS, and drinking status (*P* for trend = 0.72 and 0.52, resp.).

Incidence of MS was evaluated across the 5-year follow-up period. During this period, a total of 3,344 participants received a new diagnosis of MS. The incidence of MS was 113 per 1,000 person-years. Among the six leukocyte count groups, the respective rates of MS were 61, 86, 110, 144, 158, and 160 per 1,000 person-years. The crude and adjusted relationships between categories of leukocyte counts and the incidence of MS are indicated in Table 4. In the crude model, the unadjusted HRs (95% CI) of MS were related to the gradual increase of the categories of leukocyte counts as compared with participants who had subnormal leukocyte counts and were as follows: 1.40 (1.18, 1.66), 1.79 (1.51, 2.12), 2.34 (1.97, 2.77), 2.58 (2.15, 3.09), and 2.59 (2.13, 3.16), respectively (*P* for trend <0.0001). In the final multivariate models, the adjusted HRs (95% CI) of MS were related to the gradual increase of the categories of leukocyte counts as compared with participants who had subnormal leukocyte counts and were as follows: 1.21 (1.02, 1.44), 1.35 (1.14, 1.59), 1.58 (1.33, 1.88), 1.64 (1.36, 1.98), and 1.50 (1.22, 1.84), respectively (*P* for trend <0.0001). Moreover, after adjustment for potential confounders, the HRs (95% CI) of MS for increasing quintiles of neutrophil and lymphocyte counts were 1.00, 1.22 (1.08, 1.37), 1.30 (1.16, 1.46), 1.29 (1.15, 1.44), and 1.41 (1.25, 1.58) (*P* for trend <0.0001) and 1.00, 1.12 (1.01, 1.25), 1.21 (1.05, 1.39), 1.22 (1.10, 1.35), and 1.30 (1.16, 1.44) (*P* for trend <0.0001), respectively (Figure 2(b)).

## 4. Discussion

This study has examined the relationships between categories of leukocyte counts and MS in an apparently healthy population. Our results suggest that subnormal leukocytes counts were independently related to the lowest prevalence and incidence of MS. Similar relationships were also observed when neutrophil and lymphocyte counts were analyzed separately.

We adjusted for a number of potentially confounding factors in our analyses. Studies have shown that age and sex are simultaneously related to the incidence of MS and inflammatory status [16, 17]; therefore, we first adjusted for age and sex. However, adjustment for these factors did not significantly affect the relationship between the categories of leukocyte counts and MS, leading us to believe that the direct relationship between leukocyte count categories and MS was independent of age and sex. We next adjusted for BMI, which is an important risk factor which can cause a variety of adverse health effects [18]. Although the OR and HR for MS in higher leukocyte count categories, as compared with the subnormal leukocyte count groups, were greatly reduced, adjustment for this factor did not significantly affect the relationship between leukocyte count categories and MS. We subsequently adjusted for lifestyle factors including smoking (a major factor for leukocytes count increase [19, 20]), drinking status, and any effects of family history of diseases including CVD, hypertension, hyperlipidemia, and diabetes (which are recognized as genetic factors) of MS. However, these adjustments also did not affect the positive relationship between leukocyte count categories and MS.

Leukocyte counts are a simple, widely available, inexpensive, and well-standardized biomarker of inflammation. Several cross-sectional [6–8, 21, 22] and cohort [9–11, 23, 24] studies have suggested positive relationships between leukocyte counts and MS among populations in various countries both Asian and western. Furthermore, several cohort studies also suggest leukocyte counts as a potential predictor of all-cause mortality and CVD mortality [12, 25]. However, these studies have not definitively assessed the relationship between subnormal leukocyte counts and healthy states, therefore leaving the relationship unclear. A previous study of ours has shown that maintaining an inflammatory level (hsCRP was used as an inflammatory marker) as low as possible may potentially maintain better physical performance [26]. In the present study, we had hypothesized that the participants with subnormal leukocyte counts would have the healthiest states among apparently healthy adults. Thus, the subnormal category was used as a reference group in the analysis. In accordance with our hypothesis, the results show that the subnormal leukocyte count category had the lowest prevalence and incidence of MS.

Leukocytes are part of the body's defense against foreign substances and infections. In clinical practice, the count of leukocytes is measured as part of a routine blood test and is an important way for doctors to gauge individual health. The reference range is generally established by a 95% lower and higher limit in the peripheral blood of a general population [5]. The conventional cutoff of 4,000 cells/mm^3^ is considered a screening level for reduced leukocyte counts. However, in contrast to this general knowledge of clinical practice, we not only observed that subnormal leukocyte count levels were related to the lowest prevalence of MS in a cross-sectional study, but also found a significant relationship to the lowest incidence of MS during a 5-year follow-up period among an apparently healthy population. These findings suggest that it is necessary to restudy and discuss the clinical or preventive value of subnormal leukocyte counts among normal populations.

Based on conventional recognition, Sakuragi and his colleagues reassessed the reference values for peripheral blood leukocyte counts among the general population and attempted to examine whether the reference range had changed in the past 100 years and what its impact factors were [5]. The study concluded that leukocyte counts had a secular trend of decrease in the past 100 years with decreased smoking rates and improved general hygiene being discussed as possible factors for this reduction. Indeed, the widespread use of antibiotics and improvement of general hygiene are considered important factors for reduction of inflammatory levels; however, a more important risk factor (obesity) was not discussed in the paper. Obesity causes many immune cells to infiltrate or populate in adipose tissue and promote chronic low-grade inflammation [27]. Furthermore, fat cells, particularly those in the visceral fat, are now considered an immune organ, secreting numerous immune modulating chemicals contributing directly to the development of low-grade inflammation [28]. Over the past few decades, there has been a steep rise in the incidence of obesity worldwide. Thus, rather than general hygiene or bacterial infection, obesity has already become the strongest risk factor for a chronic inflammatory level [29, 30]. In agreement with these studies, ours also suggests that the categories of leukocyte counts were strongly and significantly related to BMI even after adjustment for age and sex. More importantly, many studies have suggested that increases in inflammatory levels related to poor hygiene and obesity may be due to a different mechanism [29, 30]. Furthermore, mounting evidence highlights the role of adipose tissue in the development of a systemic inflammatory state that contributes to vasculopathy and cardiovascular risk. Therefore, we consider it necessary to clarify the exact mechanism and health effects of leukocyte count reduction over time and then reassess its clinical or preventive value among the general population.

Our results also showed that elevated neutrophil and lymphocyte counts were independently related to the prevalence and incidence of MS. Since neutrophils are the most abundant cell type involved in the innate immune response and the cells of the adaptive immune system are lymphocytes, the results suggest that the innate and adaptive immune responses may simultaneously increase the incidence of MS. Further studies are required to clarify the hypothesis.

Nutritional status is an important risk factor for decreased leukocyte counts [31]. However, the serum albumin level, a standard indicator of nutritional status, was not significantly different among the eight categories of leukocyte counts in our study population (see Tables 1 and 3). Therefore, we deduced that nutritional status is not a major reason for the lower leukocytes count in our study population.

The present study does have a limitation in that although we adjusted for a considerable number of potentially confounding factors, we cannot exclude the possibility that MS is affected by other lifestyle variables which are intrinsically related to leukocyte counts.

## 5. Conclusions

In conclusion, this large-scale epidemiological study has shown that subnormal leukocyte counts were independently related to the lowest prevalence and incidence of MS. Based on these findings, we have constructed the novel hypothesis that a subnormal leukocyte count is not a reference value for screening cases of reduced leukocyte counts, but is maybe the healthiest range for prevention and treatment of MS or CVD among an apparently healthy population. These findings also strongly suggest that it is necessary to restudy and discuss the clinical or preventive value and the possible mechanism of subnormal leukocyte counts among the general population.

## Figures and Tables

**Table 1:** Cross-sectional analysis: age- and sex-adjusted baseline characteristics of the subjects according to categories of peripheral blood leukocyte counts (*n* = 46,179)^1^.

	Categories of peripheral blood leukocyte counts (range: ×1,000 cells/mm^3^)								*P* for trend^2^
	Subnormal (1.1–3.9)	Level 2 (4.0–4.9)	Level 3 (5.0–5.9)	Level 4 (6.0–6.9)	Level 5 (7.0–7.9)	Level 6 (8.0–8.9)	Level 7 (9.0–9.9)	Above-normal (10.0–25.6)	
	(*n* = 3556)	(*n* = 10915)	(*n* = 13883)	(*n* = 9867)	(*n* = 4789)	(*n* = 1932)	(*n* = 742)	(*n* = 495)	
Age (y)	46.0 (45.6, 46.4)^3^	45.3 (45.1, 45.5)	45.0 (44.8, 45.2)	44.8 (44.5, 45)	44.6 (44.2, 44.9)	45 (44.4, 45.6)	46.1 (45.2, 47)	44.3 (43.2, 45.4)	<0.0001
Sex (male, %)	37.2	49.4	60.4	66.8	70.6	77.0	79.8	78.4	<0.0001
BMI (kg/m^2^)	23.5 (23.4, 23.7)	24.2 (24.2, 24.3)	24.9 (24.8, 24.9)	25.4 (25.4, 25.5)	25.8 (25.7, 25.9)	26 (25.8, 26.1)	25.9 (25.7, 26.2)	25.9 (25.6, 26.2)	<0.0001
Waist circumference (cm)	80.3 (80.0, 80.6)	81.9 (81.8, 82.1)	83.6 (83.5, 83.8)	85.0 (84.8, 85.1)	86.3 (86, 86.5)	86.5 (86.1, 86.9)	86.9 (86.3, 87.5)	86.7 (85.9, 87.4)	<0.0001
TC (mmol/L)	4.99 (4.96, 5.02)	5.09 (5.07, 5.11)	5.15 (5.13, 5.16)	5.2 (5.18, 5.22)	5.23 (5.2, 5.26)	5.22 (5.18, 5.27)	5.28 (5.21, 5.35)	5.29 (5.2, 5.37)	<0.0001
TG (mmol/L)	1.22 (1.17, 1.27)	1.38 (1.35, 1.4)	1.56 (1.53, 1.58)	1.69 (1.66, 1.72)	1.89 (1.85, 1.92)	1.91 (1.85, 1.97)	2.05 (1.95, 2.14)	2.03 (1.9, 2.15)	<0.0001
LDL (mmol/L)	2.97 (2.94, 3)	3.04 (3.02, 3.05)	3.07 (3.06, 3.09)	3.11 (3.09, 3.12)	3.12 (3.09, 3.14)	3.11 (3.07, 3.14)	3.13 (3.07, 3.19)	3.13 (3.06, 3.2)	<0.0001
HDL (mmol/L)	1.52 (1.51, 1.53)	1.47 (1.47, 1.48)	1.43 (1.42, 1.43)	1.4 (1.4, 1.41)	1.38 (1.37, 1.39)	1.36 (1.35, 1.38)	1.37 (1.34, 1.39)	1.36 (1.33, 1.39)	<0.0001
SBP (mmHg)	118.8 (118.2, 119.3)	120.3 (120, 120.6)	122.1 (121.8, 122.3)	122.7 (122.4, 123.1)	123.8 (123.4, 124.3)	124.5 (123.8, 125.2)	124.8 (123.7, 126)	124.2 (122.8, 125.6)	<0.0001
DBP (mmHg)	75.4 (75.1, 75.8)	76.6 (76.4, 76.8)	77.5 (77.3, 77.7)	78.1 (77.9, 78.3)	78.7 (78.4, 79)	79 (78.6, 79.5)	79.1 (78.4, 79.9)	78.1 (77.1, 79)	<0.0001
FBS (mmol/L)	4.94 (4.9, 4.98)	4.98 (4.95, 5)	5.04 (5.02, 5.06)	5.07 (5.05, 5.09)	5.11 (5.08, 5.15)	5.14 (5.09, 5.2)	5.13 (5.05, 5.22)	5.07 (4.97, 5.17)	<0.0001
Serum UA (*μ*mol/L)	290.5 (288.2, 292.8)	299.8 (298.5, 301.2)	310.1 (308.9, 311.2)	315.4 (314, 316.8)	320.5 (318.5, 322.5)	317.8 (314.6, 321)	321.4 (316.4, 326.5)	318.6 (312.4, 324.8)	<0.0001
Fibrinogen (g/L)	2.61 (2.59, 2.63)	2.67 (2.66, 2.67)	2.68 (2.68, 2.69)	2.73 (2.72, 2.74)	2.75 (2.73, 2.76)	2.79 (2.77, 2.82)	2.85 (2.81, 2.89)	2.81 (2.76, 2.86)	<0.0001
Albumin (g/L)	46.2 (46.1, 46.3)	46.2 (46.1, 46.2)	46.2 (46.1, 46.2)	46.1 (46, 46.1)	46 (45.9, 46.1)	45.8 (45.7, 46)	45.7 (45.4, 46)	45.8 (45.5, 46.1)	0.86
Smoking status (%)									
Smoker	12.7	18.2	26.4	35.2	44.6	54.2	62.7	64.9	<0.0001
Ex-smoker	0.10	0.05	0.05	0.03	0.10	0.10	0.00	0.00	0.52
Drinker (%)	25.5	33.6	39.3	42.8	44.6	48.1	50.3	50.5	0.02
Family history of diseases (%)									
CVD	25.5	25.8	25.7	24.7	25.6	25.8	24.5	24.9	0.37
Hypertension	39.7	40.8	42.4	41.9	41.5	44.7	41.9	42.0	<0.01
Hyperlipidemia	0.67	0.54	0.60	0.72	0.58	0.52	0.54	0.00	0.38
Diabetes	14.5	15.4	16.6	16.7	17.6	19.2	21.0	19.8	<0.0001

^1^BMI: body mass index; TC: total cholesterol; TG: triglycerides; LDL: low-density lipoprotein cholesterol; HDL: high-density lipoprotein cholesterol; SBP: systolic blood pressure; DBP: diastolic blood pressure; FBS: fasting blood sugar; UA: uric acid; CVD: cardiovascular disease.

^2^Analysis of covariance or logistic regression analysis adjusted for age and sex where appropriate.

^3^Adjusted least squares mean (95% confidence interval) (all such values).

**Table 2:** Cross-sectional analysis: adjusted relationships of categories of peripheral blood leukocyte counts to metabolic syndrome (*n* = 46,179)^1^.

	Categories of peripheral blood leukocyte counts (range: ×1,000 cells/mm^3^)								*P* for trend^2^
	Subnormal (1.1–3.9)	Level 2 (4.0–4.9)	Level 3 (5.0–5.9)	Level 4 (6.0–6.9)	Level 5 (7.0–7.9)	Level 6 (8.0–8.9)	Level 7 (9.0–9.9)	Above-normal (10.0–25.6)	
	(*n* = 3556)	(*n* = 10915)	(*n* = 13883)	(*n* = 9867)	(*n* = 4789)	(*n* = 1932)	(*n* = 742)	(*n* = 495)	
Number with metabolic syndrome (presence of 3 of 5 risk factors)	514	2151	3848	3241	1824	814	325	194	—
Crude	Reference	1.45 (1.31, 1.61)^3^	2.27 (2.05, 2.51)	2.90 (2.62, 3.21)	3.64 (3.26, 4.07)	4.31 (3.79, 4.91)	4.61 (3.88, 5.48)	3.81 (3.11, 4.67)	<0.0001
Age- and sex-adjusted	Reference	1.40 (1.26, 1.56)	2.13 (1.92, 2.37)	2.68 (2.42, 2.99)	3.39 (3.03, 3.81)	3.84 (3.35, 4.40)	3.87 (3.24, 4.63)	3.43 (2.77, 4.24)	<0.0001
Age-, sex-, and BMI-adjusted	Reference	1.20 (1.07, 1.35)	1.58 (1.41, 1.77)	1.77 (1.58, 1.99)	2.07 (1.83, 2.34)	2.30 (1.99, 2.67)	2.42 (1.99, 2.93)	2.08 (1.65, 2.61)	<0.0001
Multiple adjusted^4^	Reference	1.19 (1.06, 1.34)	1.56 (1.39, 1.74)	1.74 (1.55, 1.96)	2.02 (1.78, 2.29)	2.20 (1.90, 2.56)	2.28 (1.87, 2.77)	1.98 (1.57, 2.49)	<0.0001
*Metabolic syndrome components *									
Number with elevated waist circumference (≥85 cm in males; ≥80 cm in females)	1437	5333	8200	6442	3393	1405	555	363	—
Crude	Reference	1.41 (1.31, 1.52)	2.13 (1.97, 2.29)	2.77 (2.56, 3.00)	3.58 (3.27, 3.93)	3.93 (3.49, 4.44)	4.38 (3.67, 5.24)	4.06 (3.30, 5.02)	<0.0001
Age- and sex-adjusted	Reference	1.33 (1.22, 1.45)	1.92 (1.77, 2.08)	2.44 (2.24, 2.66)	3.15 (2.85, 3.48)	3.15 (2.77, 3.59)	3.22 (2.67, 3.90)	3.32 (2.66, 4.17)	<0.0001
Age-, sex-, and BMI-adjusted	Reference	0.96 (0.86, 1.07)	1.07 (0.96, 1.20)	1.12 (0.99, 1.26)	1.31 (1.15, 1.50)	1.19 (1.00, 1.42)	1.36 (1.05, 1.75)	1.53 (1.13, 2.08)	<0.0001
Multiple adjusted^4^	Reference	0.95 (0.85, 1.07)	1.05 (0.94, 1.17)	1.08 (0.96, 1.21)	1.23 (1.07, 1.41)	1.09 (0.91, 1.30)	1.21 (0.94, 1.57)	1.35 (0.99, 1.84)	<0.0001
Number with elevated triglycerides (≥1.7 mmol/L)	497	2445	4418	3756	2114	957	385	261	—
Crude	Reference	1.78 (1.60, 1.98)	2.87 (2.60, 3.18)	3.78 (3.42, 4.20)	4.86 (4.36, 5.44)	6.04 (5.31, 6.89)	6.64 (5.59, 7.89)	6.86 (5.62, 8.39)	<0.0001
Age- and sex-adjusted	Reference	1.64 (1.48, 1.83)	2.47 (2.23, 2.74)	3.13 (2.82, 3.48)	3.96 (3.54, 4.44)	4.65 (4.08, 5.32)	4.90 (4.11, 5.85)	5.33 (4.34, 6.55)	<0.0001
Age-, sex-, and BMI-adjusted	Reference	1.49 (1.33, 1.66)	2.02 (1.82, 2.25)	2.37 (2.13, 2.65)	2.84 (2.53, 3.20)	3.29 (2.87, 3.78)	3.54 (2.95, 4.25)	3.90 (3.15, 4.83)	<0.0001
Multiple adjusted^4^	Reference	1.48 (1.33, 1.65)	1.99 (1.79, 2.21)	2.29 (2.06, 2.56)	2.68 (2.39, 3.02)	3.03 (2.64, 3.49)	3.17 (2.63, 3.81)	3.49 (2.82, 4.33)	<0.0001
Number with reduced HDL (<1.0 mmol/L in males; <1.3 mmol/L in females)	576	1799	2611	2005	1083	487	180	130	—
Crude	Reference	1.04 (0.94, 1.15)	1.22 (1.11, 1.35)	1.34 (1.21, 1.49)	1.54 (1.38, 1.73)	1.78 (1.55, 2.04)	1.69 (1.39, 2.04)	1.88 (1.5, 2.33)	<0.0001
Age- and sex-adjusted	Reference	1.14 (1.02, 1.26)	1.46 (1.32, 1.61)	1.7 (1.53, 1.88)	2.01 (1.79, 2.26)	2.47 (2.14, 2.84)	2.41 (1.98, 2.92)	2.63 (2.1, 3.28)	<0.0001
Age-, sex-, and BMI-adjusted	Reference	1.06 (0.95, 1.18)	1.27 (1.15, 1.41)	1.38 (1.24, 1.54)	1.57 (1.4, 1.77)	1.9 (1.65, 2.19)	1.85 (1.52, 2.26)	2.06 (1.63, 2.57)	<0.0001
Multiple adjusted^4^	Reference	1.06 (0.96, 1.18)	1.25 (1.13, 1.39)	1.34 (1.21, 1.5)	1.49 (1.33, 1.68)	1.76 (1.52, 2.03)	1.67 (1.37, 2.04)	1.84 (1.46, 2.31)	<0.0001
Number with elevated blood pressure (systolic: ≥130 and/or diastolic: ≥85 mmHg)	1116	3952	5744	4332	2219	934	388	232	—
Crude	Reference	1.24 (1.15, 1.35)	1.54 (1.43, 1.67)	1.71 (1.58, 1.86)	1.89 (1.72, 2.07)	2.05 (1.83, 2.29)	2.40 (2.04, 2.81)	1.93 (1.59, 2.33)	<0.0001
Age- and sex-adjusted	Reference	1.23 (1.13, 1.34)	1.51 (1.38, 1.64)	1.65 (1.51, 1.81)	1.83 (1.66, 2.02)	1.88 (1.66, 2.13)	2.06 (1.73, 2.45)	1.81 (1.48, 2.23)	<0.0001
Age-, sex-, and BMI-adjusted	Reference	1.10 (1.00, 1.20)	1.19 (1.09, 1.30)	1.20 (1.09, 1.31)	1.23 (1.11, 1.37)	1.24 (1.09, 1.41)	1.39 (1.16, 1.66)	1.21 (0.98, 1.50)	<0.0001
Multiple adjusted^4^	Reference	1.10 (1.00, 1.20)	1.21 (1.11, 1.33)	1.25 (1.14, 1.37)	1.34 (1.21, 1.49)	1.36 (1.19, 1.55)	1.59 (1.33, 1.90)	1.40 (1.13, 1.73)	<0.0001
Number with elevated fasting glucose (≥5.56 mmol/L)	426	1536	2348	1779	968	426	175	105	—
Crude	Reference	1.2 (1.07, 1.35)	1.5 (1.34, 1.67)	1.62 (1.44, 1.81)	1.86 (1.65, 2.11)	2.08 (1.79, 2.41)	2.27 (1.86, 2.76)	1.98 (1.55, 2.5)	<0.0001
Age- and sex-adjusted	Reference	1.17 (1.04, 1.32)	1.41 (1.26, 1.58)	1.49 (1.33, 1.68)	1.73 (1.52, 1.97)	1.85 (1.59, 2.16)	1.91 (1.56, 2.35)	1.81 (1.41, 2.31)	<0.0001
Age-, sex-, and BMI-adjusted	Reference	1.07 (0.95, 1.21)	1.17 (1.05, 1.32)	1.17 (1.04, 1.32)	1.29 (1.13, 1.47)	1.35 (1.16, 1.58)	1.42 (1.15, 1.74)	1.37 (1.07, 1.76)	<0.0001
Multiple adjusted^4^	Reference	1.06 (0.94, 1.19)	1.16 (1.03, 1.3)	1.16 (1.03, 1.31)	1.28 (1.12, 1.47)	1.33 (1.14, 1.56)	1.37 (1.11, 1.69)	1.33 (1.03, 1.71)	<0.0001

^1^BMI: body mass index; HDL: high-density lipoprotein cholesterol.

^2^Multiple logistic regression analysis.

^3^Unadjusted odds ratios (95% confidence interval) (all such values).

^4^Adjusted for age, sex, BMI, smoking status, drinking status, and family history of cardiovascular disease, hypertension, hyperlipidemia, and diabetes.

(Metabolic syndrome was defined with AHA Scientific Statements criteria in 2009 [15].)

**Table 3:** Cohort analysis: age- and sex-adjusted baseline characteristics of the subjects according to categories of peripheral blood leukocyte counts (*n* = 13,061)^1^.

	Categories of peripheral blood leukocyte counts (range: ×1,000 cells/mm^3^)								*P* for trend^2^
	Subnormal (1.1–3.9)	Level 2 (4.0–4.9)	Level 3 (5.0–5.9)	Level 4 (6.0–6.9)	Level 5 (7.0–7.9)	Level 6 (8.0–8.9)	Level 7 (9.0–9.9)	Above-normal (10.0–14.5)	
	(*n* = 1123)	(*n* = 3391)	(*n* = 4019)	(*n* = 2636)	(*n* = 1182)	(*n* = 442)	(*n* = 163)	(*n* = 105)	
Age (y)	44.8 (44.0, 45.5)^3^	43.9 (43.5, 44.3)	43.1 (42.7, 43.5)	43.0 (42.6, 43.5)	42.5 (41.8, 43.2)	43.9 (43.0, 44.8)			<0.0001
Sex (male, %)	36.8	49.2	58.9	64.4	66.6	75.2			<0.0001
BMI (kg/m^2^)	23.0 (22.8, 23.2)	23.5 (23.4, 23.6)	24.1 (24.0, 24.2)	24.6 (24.4, 24.7)	24.8 (24.7, 25.0)	24.8 (24.6, 25.1)			<0.0001
Waist circumference (cm)	78.1 (77.6, 78.5)	79.4 (79.2, 79.7)	80.9 (80.7, 81.2)	82.0 (81.6, 82.3)	82.8 (82.3, 83.2)	83.1 (82.5, 83.6)			<0.0001
TC (mmol/L)	4.94 (4.88, 4.99)	5.00 (4.97, 5.03)	5.03 (5.00, 5.06)	5.07 (5.03, 5.10)	5.07 (5.02, 5.12)	5.08 (5.01, 5.14)			<0.01
TG (mmol/L)	1.00 (0.95, 1.05)	1.11 (1.08, 1.13)	1.23 (1.20, 1.25)	1.31 (1.28, 1.34)	1.36 (1.31, 1.41)	1.47 (1.41, 1.52)			<0.0001
LDL (mmol/L)	2.94 (2.89, 2.98)	2.99 (2.97, 3.02)	3.01 (2.99, 3.04)	3.05 (3.02, 3.08)	3.06 (3.01, 3.10)	3.01 (2.95, 3.07)			<0.01
HDL (mmol/L)	1.58 (1.56, 1.59)	1.54 (1.53, 1.55)	1.50 (1.49, 1.51)	1.47 (1.45, 1.48)	1.43 (1.41, 1.45)	1.45 (1.43, 1.47)			<0.0001
SBP (mmHg)	115.2 (114.4, 116)	116.3 (115.8, 116.8)	117.5 (117.1, 117.9)	117.5 (116.9, 118.0)	118.2 (117.4, 119.0)	117.4 (116.4, 118.4)			<0.0001
DBP (mmHg)	73.3 (72.7, 73.8)	74.1 (73.8, 74.4)	74.8 (74.6, 75.1)	75.2 (74.9, 75.6)	75.3 (74.8, 75.8)	75.3 (74.6, 76.0)			<0.0001
FBS (mmol/L)	4.75 (4.71, 4.79)	4.76 (4.74, 4.79)	4.76 (4.73, 4.78)	4.75 (4.73, 4.78)	4.71 (4.67, 4.75)	4.69 (4.64, 4.75)			0.72
Serum UA (*μ*mol/L)	283.2 (279.4, 286.9)	290.3 (288.2, 292.5)	299.0 (297.0, 301.0)	302.2 (299.7, 304.6)	305.6 (301.9, 309.3)	303.1 (298.4, 307.9)			<0.0001
Fibrinogen (g/L)	2.63 (2.6, 2.66)	2.67 (2.65, 2.68)	2.68 (2.66, 2.69)	2.71 (2.69, 2.73)	2.72 (2.69, 2.75)	2.80 (2.77, 2.84)			<0.01
Albumin (g/L)	46.0 (45.8, 46.2)	46.0 (45.9, 46.1)	45.9 (45.8, 46.0)	45.7 (45.6, 45.9)	45.7 (45.5, 45.9)	45.4 (45.1, 45.6)			0.41
Smoking status (%)									
Smoker	11.0	16.6	22.6	31.1	40.5	54.9			<0.0001
Ex-smoker	0.00	0.03	0.02	0.00	0.00	0.14			0.23
Drinker (%)	19.8	26.7	32.7	35.2	36.9	43.2			<0.001
Family history of diseases (%)									
CVD	35.5	34.8	33.3	31.7	33.3	34.1			0.74
Hypertension	49.0	47.4	49.5	48.8	47.6	48.2			0.52
Hyperlipidemia	1.25	1.18	1.05	1.14	0.76	0.56			0.16
Diabetes	15.9	17.5	18.5	19.7	21.0	22.7			<0.0001

^1^BMI: body mass index; TC: total cholesterol; TG: triglycerides; LDL: low-density lipoprotein cholesterol; HDL: high-density lipoprotein cholesterol; SBP: systolic blood pressure; DBP: diastolic blood pressure; FBS: fasting blood sugar; UA: uric acid; CVD: cardiovascular disease.

^2^Analysis of covariance or logistic regression analysis adjusted for age and sex where appropriate.

^3^Adjusted least squares mean (95% confidence interval) (all such values).

**Table 4:** Cohort analysis: adjusted relationships of categories of peripheral blood leukocyte counts to the incidence of the metabolic syndrome (*n* = 13,061)^1^.

	Categories of peripheral blood leukocyte counts (range: ×1,000 cells/mm^3^)								*P* for trend^2^
	Subnormal (1.1–3.9)	Level 2 (4.0–4.9)	Level 3 (5.0–5.9)	Level 4 (6.0–6.9)	Level 5 (7.0–7.9)	Level 6 (8.0–8.9)	Level 7 (9.0–9.9)	Above-normal (10.0–14.5)	
	(*n* = 1123)	(*n* = 3391)	(*n* = 4019)	(*n* = 2636)	(*n* = 1182)	(*n* = 442)	(*n* = 163)	(*n* = 105)	
Person-years of follow-up	2575	7724	9172	5811	2654	979	394	249	—
Number with metabolic syndrome (presence of 3 of 5 risk factors)	158	662	1010	835	420	162	58	39	—
Crude	Reference	1.40 (1.18, 1.66)^3^	1.79 (1.51, 2.12)	2.34 (1.97, 2.77)	2.58 (2.15, 3.09)	2.59 (2.13, 3.16)			<0.0001
Age- and sex-adjusted	Reference	1.32 (1.11, 1.58)	1.63 (1.38, 1.93)	2.07 (1.74, 2.46)	2.28 (1.90, 2.74)	2.15 (1.76, 2.63)			<0.0001
Age-, sex-, and BMI-adjusted	Reference	1.22 (1.03, 1.45)	1.36 (1.14, 1.60)	1.60 (1.34, 1.90)	1.67 (1.39, 2.02)	1.56 (1.28, 1.91)			<0.0001
Multiple adjusted^4^	Reference	1.21 (1.02, 1.44)	1.35 (1.14, 1.59)	1.58 (1.33, 1.88)	1.64 (1.36, 1.98)	1.50 (1.22, 1.84)			<0.0001

^1^BMI: body mass index; HDL: high-density lipoprotein cholesterol.

^2^Analysis by Cox proportional hazards model.

^3^Unadjusted hazard ratios (95% confidence interval) (all such values).

^4^Adjusted for age, sex, BMI, smoking status, drinking status, and family history of cardiovascular disease, hypertension, hyperlipidemia, and diabetes.

(Metabolic syndrome was defined with AHA Scientific Statements criteria in 2009 [15].)
